# Bis(4-fluoro­anilinium) tetra­chloridocuprate(II)

**DOI:** 10.1107/S1600536810017289

**Published:** 2010-05-15

**Authors:** Min Min Zhao, Ping Ping Shi

**Affiliations:** a Ordered Matter Science Research Center, College of Chemistry and Chemical Engineering, Southeast University, Nanjing 211189, People’s Republic of China

## Abstract

The crystal structure of the title compound, (C_6_H_7_FN)_2_[CuCl_4_], consists of parallel two-dimensional perovskite-type layers of corner-sharing CuCl_6_ octa­hedra. These are bonded together *via* N—H⋯Cl hydrogen bonds from the 4-fluoro­anilinium chains, which are almost perpendicular to the layers. The CuCl_4_ dianions have two short Cu—Cl bonds [2.2657 (15) and 2.2884 (13) Å] and two longer bonds [2.8868 (15) Å], giving highly Jahn–Teller-distorted CuCl_6_ octa­hedra. The Cu atoms are situated on crystallographic centers of inversion.

## Related literature

For similar ammonium salts, see: Yuan *et al.* (2004 ▶); Bhattacharya *et al.* (2004 ▶). For the ferroelectric properties of a related ammonium metal(II) salt, see: Zhang *et al.* (2009 ▶); Ye *et al.* (2009 ▶).
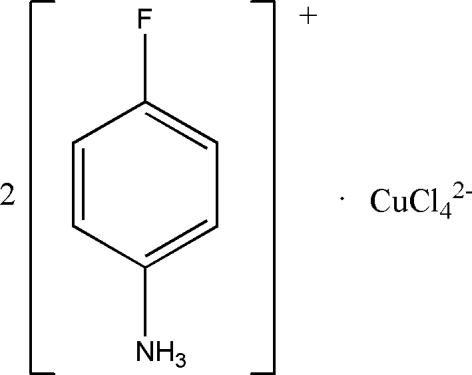


## Experimental

### 

#### Crystal data

(C_6_H_7_FN)_2_[CuCl_4_]
*M* *_r_* = 429.59
Monoclinic, 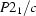

*a* = 15.603 (3) Å
*b* = 7.3893 (15) Å
*c* = 7.1238 (14) Å
β = 99.92 (3)°
*V* = 809.0 (3) Å^3^
*Z* = 2
Mo *K*α radiation
μ = 2.02 mm^−1^
*T* = 293 K
0.20 × 0.20 × 0.20 mm

#### Data collection

Rigaku SCXmini diffractometer
Absorption correction: multi-scan (*CrystalClear*; Rigaku, 2005 ▶) *T*_min_ = 0.667, *T*_max_ = 0.674
8010 measured reflections
1863 independent reflections
1555 reflections with *I* > 2σ(*I*)
*R*_int_ = 0.050

#### Refinement

*R*[*F* ^2^ > 2σ(*F* ^2^)] = 0.058
*wR*(*F* ^2^) = 0.166
*S* = 1.16
1863 reflections
98 parameters
H-atom parameters constrained
Δρ_max_ = 1.03 e Å^−3^
Δρ_min_ = −0.88 e Å^−3^

### 

Data collection: *CrystalClear* (Rigaku, 2005 ▶); cell refinement: *CrystalClear*; data reduction: *CrystalClear*; program(s) used to solve structure: *SHELXS97* (Sheldrick, 2008 ▶); program(s) used to refine structure: *SHELXL97* (Sheldrick, 2008 ▶); molecular graphics: *SHELXTL* (Sheldrick, 2008 ▶); software used to prepare material for publication: *PRPKAPPA* (Ferguson, 1999 ▶).

## Supplementary Material

Crystal structure: contains datablocks I, global. DOI: 10.1107/S1600536810017289/im2199sup1.cif

Structure factors: contains datablocks I. DOI: 10.1107/S1600536810017289/im2199Isup2.hkl

Additional supplementary materials:  crystallographic information; 3D view; checkCIF report

## Figures and Tables

**Table 1:** Hydrogen-bond geometry (Å, °)

*D*—H⋯*A*	*D*—H	H⋯*A*	*D*⋯*A*	*D*—H⋯*A*
N1—H1*B*⋯Cl2	0.89	2.37	3.248 (6)	168
N1—H1*A*⋯Cl3^i^	0.89	2.37	3.196 (5)	154
N1—H1*C*⋯Cl3^ii^	0.89	2.55	3.353 (6)	151

Symmetry codes: (i) 
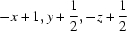
; (ii) 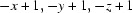.

**Fig. 1.**
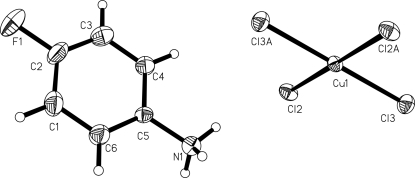
The molecular structure of one cation and one anion of the title compound, with the atomic numbering scheme. Displacement ellipsoids are drawn at the 30% probability level.

**Fig. 2.**
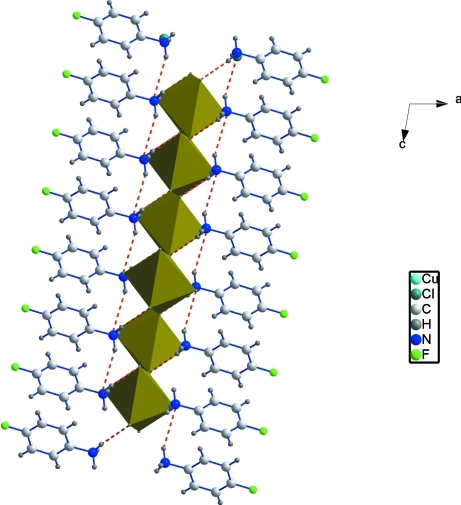
A view of the packing of the title compound, stacking along the b axis. Dashed lines indicate hydrogen bonds.

## supplementary crystallographic information

### Comment

Copper(II) halides occur in a variety of geometrical conformations including tetrahedral, square-pyramidal, square-bipyramidal, square-planar and trigonal–bipyramidal (Bhattacharya *et al.*, 2004; Yuan *et al.*, 2004). The perovskite-layer copper chlorides have attracted a great deal of attention due to their magnetic properties and interesting structural phase transitions. This study is a part of our systematic investigation of dielectric ferroelectric, phase transitions materials (Ye *et al.*, 2009; Zhang *et al.*, 2009), including organic ligands, metal-organic coordination compounds and organic inorganic hybrid compounds. Below the melting point (m.p. 440 K) of the 4-fluoroanilinium tetrachlorocuprate, the dielectric constant as a function of temperature also goes smoothly, and there is no dielectric anomaly observed (dielectric constant equaling to 6 to 11).

The asymmetric unit of the title compound is composed of a (C_6_H_7_FN^+^) cation and one half of the anionic (CuCl_4_^2-^) moiety (Fig 1). Tetrachlorocuprate(II) salt of 4-fluoroanilinium ion typically crystallizes in a two-dimensional perovskite-type (CuCl_4_^2-^) layer structure with layers separated by the organic cations. The CuCl_4_^2-^ ion is almost square, with an out-of-plane Cu1—Cl3 bond length of 2.266 (2) Å , an in-plane Cu1—Cl2 bond length of 2.288 (1) Å and a Cl3—Cu1—Cl2 angle of 90.06 (6)°. The perovskite-type layer consists of cornersharing octahedra in the *bc* plane. The distance of Cu to the in-plane Cl2 atom of the next CuCl_4_^2-^ ion is approximately 2.9 Å and is significantly longer than the distances in the CuCl_4_^2-^ square due to the Jahn-Teller effect. The Cu atom is situated on a crystallographic center of inversion. In the *bc* plane, Cu atoms and Cl2 atoms form a puckered plane and the Cu—Cl3 bond is nearly perpendicular to this plane. The organic chains are arranged between the layers. NH_3_^+^ groups fit into cavities of the CuCl_4_^2-^ layer and N—H···Cl hydrogen bonds bind the organic chains (Fig. 2). Details of the hydrogen-bonding geometry are given in Table 1.

### Experimental

An excess of hydrogen chloride was slowly added to 20 ml of an ethanolic solution of 4-fluoroaniline (222 mg, 0.002 mol). Then copper dichloride dihydrate (170 mg, 0.001 mol) was added to the mixture. After several days, the title salt, (C_6_H_7_FN^+)^_2_(CuCl_4_^2-^), was formed and recrystallized from an ethanolic solution at room temperature to afford green prismatic crystals suitable for X-ray analysis.

Dielectric studies (capacitance and dielectric loss measurements) were performed on powder samples which have been pressed into tablets on the surfaces of which a conducting carbon glue was deposited. The automatic impedance TongHui2828 Analyzer has been used. In the measured temperature ranges (80 K to 430 K), the title structure showed no dielectric anomaly.

### Refinement

All C—H hydrogen atoms were calculated geometrically and were refined using a riding model with C—H distances ranging from 0.93 to 0.97 Å and *U*_iso_(H) = 1.2 U_eq_(C). Hydrogen positions at nitrogen were also calculated geometrically and included into the refinement with N—H = 0.89 Å and *U*_i__so_(H) = 1.5 U_eq_(N).

### Crystal data

**Table:**

(C_6_H_7_FN)_2_[CuCl_4_]	*F*(000) = 430
*M**_r_* = 429.59	*D*_x_ = 1.763 Mg m^−^^3^
Monoclinic, *P*2_1_/*c*	Mo *K*α radiation, λ = 0.71073 Å
Hall symbol: -P 2ybc	Cell parameters from 7279 reflections
*a* = 15.603 (3) Å	θ = 3.1–27.5°
*b* = 7.3893 (15) Å	µ = 2.02 mm^−^^1^
*c* = 7.1238 (14) Å	*T* = 293 K
β = 99.92 (3)°	Prism, green
*V* = 809.0 (3) Å^3^	0.20 × 0.20 × 0.20 mm
*Z* = 2	

### Data collection

**Table:**

Rigaku SCXmini diffractometer	1863 independent reflections
Radiation source: fine-focus sealed tube	1555 reflections with *I* > 2σ(*I*)
graphite	*R*_int_ = 0.050
Detector resolution: 13.6612 pixels mm^-1^	θ_max_ = 27.5°, θ_min_ = 3.1°
CCD_Profile_fitting scans	*h* = −20→20
Absorption correction: multi-scan (*CrystalClear*; Rigaku, 2005)	*k* = −9→9
*T*_min_ = 0.667, *T*_max_ = 0.674	*l* = −9→9
8010 measured reflections	

### Refinement

**Table:**

Refinement on *F*^2^	Primary atom site location: structure-invariant direct methods
Least-squares matrix: full	Secondary atom site location: difference Fourier map
*R*[*F*^2^ > 2σ(*F*^2^)] = 0.058	Hydrogen site location: inferred from neighbouring sites
*wR*(*F*^2^) = 0.166	H-atom parameters constrained
*S* = 1.16	*w* = 1/[σ^2^(*F*_o_^2^) + (0.059*P*)^2^ + 3.9072*P*] where *P* = (*F*_o_^2^ + 2*F*_c_^2^)/3
1863 reflections	(Δ/σ)_max_ = 0.001
98 parameters	Δρ_max_ = 1.02 e Å^−^^3^
0 restraints	Δρ_min_ = −0.88 e Å^−^^3^

### Special details

**Table:**

Geometry. All esds (except the esd in the dihedral angle between two l.s. planes) are estimated using the full covariance matrix. The cell esds are taken into account individually in the estimation of esds in distances, angles and torsion angles; correlations between esds in cell parameters are only used when they are defined by crystal symmetry. An approximate (isotropic) treatment of cell esds is used for estimating esds involving l.s. planes.
Refinement. Refinement of *F*^2^ against ALL reflections. The weighted *R*-factor wR and goodness of fit *S* are based on *F*^2^, conventional *R*-factors *R* are based on *F*, with *F* set to zero for negative *F*^2^. The threshold expression of *F*^2^ > σ(*F*^2^) is used only for calculating *R*-factors(gt) etc. and is not relevant to the choice of reflections for refinement. *R*-factors based on *F*^2^ are statistically about twice as large as those based on *F*, and *R*- factors based on ALL data will be even larger.

### Fractional atomic coordinates and isotropic or equivalent isotropic displacement parameters (Å^2^)

**Table:**

	*x*	*y*	*z*	*U*_iso_*/*U*_eq_	
C1	0.1209 (5)	0.6057 (12)	0.4232 (12)	0.0590 (19)	
H1	0.0831	0.6781	0.4781	0.071*	
C2	0.0896 (5)	0.4848 (12)	0.2821 (12)	0.061 (2)	
C3	0.1426 (5)	0.3721 (12)	0.2064 (12)	0.066 (2)	
H3	0.1195	0.2860	0.1166	0.079*	
C4	0.2304 (4)	0.3856 (10)	0.2627 (10)	0.0487 (16)	
H4	0.2677	0.3125	0.2076	0.058*	
C5	0.2632 (4)	0.5085 (8)	0.4019 (8)	0.0333 (12)	
C6	0.2098 (4)	0.6174 (10)	0.4819 (10)	0.0486 (16)	
H6	0.2328	0.6998	0.5759	0.058*	
N1	0.3577 (3)	0.5283 (7)	0.4557 (8)	0.0392 (12)	
H1A	0.3728	0.6415	0.4337	0.059*	
H1B	0.3841	0.4524	0.3872	0.059*	
H1C	0.3735	0.5032	0.5790	0.059*	
F1	0.0021 (3)	0.4721 (10)	0.2268 (10)	0.100 (2)	
Cu1	0.5000	0.5000	0.0000	0.0265 (3)	
Cl2	0.47957 (10)	0.28942 (18)	0.22368 (19)	0.0370 (4)	
Cl3	0.64585 (9)	0.4598 (2)	0.0752 (2)	0.0403 (4)	

### Atomic displacement parameters (Å^2^)

**Table:**

	*U*^11^	*U*^22^	*U*^33^	*U*^12^	*U*^13^	*U*^23^
C1	0.042 (4)	0.066 (5)	0.071 (5)	0.009 (3)	0.015 (4)	−0.003 (4)
C2	0.033 (3)	0.087 (6)	0.058 (5)	−0.012 (4)	−0.003 (3)	0.009 (4)
C3	0.055 (5)	0.074 (6)	0.067 (5)	−0.018 (4)	0.003 (4)	−0.024 (4)
C4	0.044 (4)	0.049 (4)	0.053 (4)	−0.008 (3)	0.010 (3)	−0.015 (3)
C5	0.034 (3)	0.034 (3)	0.029 (3)	0.003 (2)	−0.001 (2)	0.003 (2)
C6	0.045 (4)	0.046 (4)	0.054 (4)	−0.001 (3)	0.006 (3)	−0.006 (3)
N1	0.043 (3)	0.033 (3)	0.041 (3)	−0.001 (2)	0.008 (2)	0.003 (2)
F1	0.037 (3)	0.143 (6)	0.114 (5)	−0.019 (3)	−0.007 (3)	−0.020 (4)
Cu1	0.0304 (5)	0.0262 (5)	0.0233 (5)	0.0012 (4)	0.0060 (3)	0.0062 (3)
Cl2	0.0525 (9)	0.0308 (7)	0.0289 (7)	−0.0017 (6)	0.0101 (6)	0.0064 (5)
Cl3	0.0301 (7)	0.0449 (8)	0.0455 (8)	0.0038 (6)	0.0050 (6)	0.0011 (6)

### Geometric parameters (Å, °)

**Table:**

C1—C2	1.370 (12)	C5—N1	1.465 (8)
C1—C6	1.381 (10)	C6—H6	0.9300
C1—H1	0.9300	N1—H1A	0.8900
C2—C3	1.350 (12)	N1—H1B	0.8900
C2—F1	1.359 (9)	N1—H1C	0.8900
C3—C4	1.362 (10)	Cu1—Cl3	2.2657 (15)
C3—H3	0.9300	Cu1—Cl3^i^	2.2657 (15)
C4—C5	1.376 (8)	Cu1—Cl2	2.2884 (13)
C4—H4	0.9300	Cu1—Cl2^i^	2.2884 (13)
C5—C6	1.353 (9)		
			
C2—C1—C6	118.3 (7)	C5—C6—C1	119.7 (7)
C2—C1—H1	120.8	C5—C6—H6	120.2
C6—C1—H1	120.8	C1—C6—H6	120.2
C3—C2—F1	119.7 (8)	C5—N1—H1A	109.5
C3—C2—C1	122.0 (7)	C5—N1—H1B	109.5
F1—C2—C1	118.1 (8)	H1A—N1—H1B	109.5
C2—C3—C4	119.4 (7)	C5—N1—H1C	109.5
C2—C3—H3	120.3	H1A—N1—H1C	109.5
C4—C3—H3	120.3	H1B—N1—H1C	109.5
C3—C4—C5	119.4 (7)	Cl3—Cu1—Cl3^i^	180.00 (2)
C3—C4—H4	120.3	Cl3—Cu1—Cl2	90.06 (6)
C5—C4—H4	120.3	Cl3^i^—Cu1—Cl2	89.94 (6)
C6—C5—C4	121.1 (6)	Cl3—Cu1—Cl2^i^	89.94 (6)
C6—C5—N1	119.7 (5)	Cl3^i^—Cu1—Cl2^i^	90.06 (6)
C4—C5—N1	119.2 (6)	Cl2—Cu1—Cl2^i^	180.00 (5)

Symmetry codes: (i) −*x*+1, −*y*+1, −*z*.

### Hydrogen-bond geometry (Å, °)

**Table:**

*D*—H···*A*	*D*—H	H···*A*	*D*···*A*	*D*—H···*A*
N1—H1B···Cl2	0.89	2.37	3.248 (6)	168
N1—H1A···Cl3^ii^	0.89	2.37	3.196 (5)	154
N1—H1C···Cl3^iii^	0.89	2.55	3.353 (6)	151

Symmetry codes: (ii) −*x*+1, *y*+1/2, −*z*+1/2; (iii) −*x*+1, −*y*+1, −*z*+1.
